# The Relationship Between Cough Reflex Sensitivity and Exacerbation Frequency in Chronic Obstructive Pulmonary Disease

**DOI:** 10.1007/s00408-020-00366-x

**Published:** 2020-06-19

**Authors:** Peter S. P. Cho, Hannah V. Fletcher, Richard D. Turner, Irem S. Patel, Caroline J. Jolley, Surinder S. Birring

**Affiliations:** 1 Centre for Human and Applied Physiological Sciences, School of Basic and Medical Biosciences, King’s College London, London, UK; 2 Department of Respiratory Medicine, King’s College Hospital NHS Foundation Trust, London, UK; 3 Department of Respiratory Medicine, Charing Cross Hospital, Imperial College Healthcare Trust, London, UK

**Keywords:** Cough reflex sensitivity, Cough, Chronic obstructive pulmonary disease, Exacerbation of chronic obstructive pulmonary disease

## Abstract

**Background:**

Cough is predictive of exacerbations of chronic obstructive pulmonary disease (COPD). Little is known about cough reflex sensitivity during exacerbation of COPD and whether it is associated with exacerbation frequency. This pilot study aimed to investigate cough reflex sensitivity during and following recovery from exacerbation of COPD, and its association with the frequency of future exacerbations. In addition, the repeatability of cough reflex sensitivity in stable COPD was investigated.

**Methods:**

Twenty participants hospitalised with exacerbation of COPD underwent inhaled capsaicin challenge during exacerbation and after 6 weeks of recovery. The frequency of future exacerbations was monitored for 12 months. The repeatability of cough reflex sensitivity was assessed in separate participants with stable COPD, who underwent 2 capsaicin challenge tests, 6 weeks apart.

**Results:**

Cough reflex sensitivity was heightened during exacerbation of COPD. Geometric mean (SD) capsaicin concentration thresholds to elicit 5 coughs (C5) during exacerbation and after 6 weeks of recovery were 1.76 (3.73) vs. 8.09 (6.25) μmol L^−1^, respectively (*p* < 0.001). The change in C5 from exacerbation to 6-week recovery was associated with the frequency of future exacerbations (*ρ* = − 0.687, *p* = 0.003). C5 was highly repeatable over 6 weeks in stable COPD, and intraclass correlation coefficient was 0.85.

**Conclusion:**

Cough reflex sensitivity is heightened during exacerbation of COPD and reduces after recovery. The persistence of cough reflex hypersensitivity at recovery was associated with the frequency of future exacerbations.

**Electronic supplementary material:**

The online version of this article (10.1007/s00408-020-00366-x) contains supplementary material, which is available to authorised users.

## Introduction

Chronic obstructive pulmonary disease (COPD) exacerbations are acute episodes of sustained deterioration in respiratory symptoms beyond normal daily variation [1]. Such episodes are associated with impaired quality of life [2], accelerated decline in lung function [3] and increased health care utilisation [4]. The identification of individuals with COPD who are more susceptible to exacerbation may lead to targeted preventative therapies. Increased cough and/or sputum volume or purulence are common symptoms of COPD exacerbations [5, 6]. Independent authors have reported that higher levels of both cough symptoms and sputum production in the stable state are associated with exacerbation frequency [2, 7, 8].

The mechanism of cough in general is complex and not fully understood [9, 10]. One potential mechanism is the activation of bronchopulmonary C fibres [9]. C fibres are relatively quiescent in health but can be activated or sensitised by inflammatory mediators [11, 12]. For example, cough reflex sensitivity to capsaicin, a potent activator of C fibres, is heightened during viral upper respiratory tract infection [13, 14]. The changes in cough reflex sensitivity during acute exacerbation of COPD have not previously been investigated.

The aim of this pilot study was, firstly, to determine if cough reflex sensitivity is heightened during exacerbation of COPD and, secondly, to investigate an association between cough reflex sensitivity and exacerbation frequency. Thirdly, we sought to determine the repeatability of cough reflex sensitivity measurements in patients with stable COPD.

## Methods

This was a prospective observational study of participants hospitalised with exacerbation of COPD (winter seasons of 2017/2018) and participants with stable COPD (April to August 2017), conducted at a single site (King’s College Hospital, Denmark Hill, London, UK) in accordance with the principles of the Declaration of Helsinki. The study was granted research ethics committee approval (Health Research Authority South East Scotland Research Ethics Service, 16/SS/0189). All participants provided written informed consent.

## Participants

### COPD Exacerbations

An exacerbation of COPD was defined as an episode of acute deterioration of respiratory symptoms associated with physiological deterioration which required additional therapy [5]. Consecutive patients admitted to hospital with an exacerbation of COPD were recruited prospectively. Initial management was according to local guidelines, with oral corticosteroids, and a combination of antibiotics, nebulised bronchodilator, inhaled meter-dosed bronchodilators and non-invasive ventilation as appropriate. Exclusion criteria were prior exposure to capsaicin challenge test, consolidation on chest radiograph, significant cognitive impairment and any additional causes for hospitalisation.

### Stable COPD (Repeatability)

To investigate repeatability, a separate group of consecutive patients with COPD and stable symptoms in the preceding 6 weeks were recruited prospectively from an outpatient specialist clinic. The diagnostic criteria were a ≥ 10 pack-year smoking history and post-bronchodilator forced expiratory volume in 1 s (FEV_1_) to forced vital capacity (FVC) ratio < 0.70 [15]. Exclusion criteria were previous exposure to capsaicin challenge test and a history suggestive of respiratory tract infection within the previous 6 weeks.

## Protocol

All participants underwent investigations at two timepoints, six weeks apart. At timepoint 1, demographic and anthropometric data were collected, and participants underwent spirometry, inhaled capsaicin tussive challenge and 24-h cough frequency monitoring. Participant-reported subjective assessments of COPD health status, cough severity and cough-specific health status were also performed. At timepoint 2, all previous measures from timepoint 1 were repeated, and the participants also underwent body plethysmography and measurements of carbon monoxide transfer factor coefficient.

### COPD Exacerbation

Twenty participants underwent investigations on the day of hospital discharge following admission for exacerbation of COPD (*T*_E_), and after a 6-week recovery period (*T*_R_). Medical fitness for discharge was determined by the senior attending physician [16]. Seventeen participants were then followed up by review of National Health Service electronic patient records for a period of 12 months to measure the frequency of further COPD exacerbations. Exacerbations were defined as episodes of healthcare use for their COPD with a change in medication or hospital admission as per Hurst et al. [17]. The diagnostic criteria used met the definition of moderate and severe exacerbations of COPD, exacerbations requiring a change of medication and to seek medical assistance and exacerbations requiring hospitalisation, respectively [18]. The primary and secondary care clinicians treated patients according to local clinical guidelines. Episodes of subsequent exacerbations of COPD, separated by an exacerbation-free period of ≥ 14 days, were determined from prospective review of the combined local hospital and primary care electronic patient record system [5]. All primary care practices in the local catchment area were linked to accessible electronic local care records.

### Repeatability of Tussive Challenge Testing in Stable COPD

Ten separate participants with stable COPD underwent investigations over 2 visits separated by 6 weeks: *T*_0_ and *T*_6_, respectively.

Any participants with either exacerbation of COPD or stable COPD, who had a respiratory tract infection, an exacerbation of COPD or a change in smoking status between the 2 visits, were excluded from data analysis.

## Capsaicin Tussive Challenge Test

Cough reflex sensitivity (CRS) was assessed according to the European Respiratory Society (ERS) guidelines [19]. Capsaicin solution (Sigma-Aldrich, Missouri, USA) was delivered in incremental doubling doses (0.49–1000 µmol L^−1^) at 1-min intervals, as 10-µL single breath inhalations, via an air-powered dosimeter (KoKo Digidoser, nSpire Health Inc, Longmont, CO, USA). To diminish the effect of anticipation, 0.9% saline solution was interspersed randomly [19]. A single characterised nebuliser (DeVilbiss Healthcare, Port Washington, NY, USA) with an output of 1.232 mL min^−1^ was employed for all participants throughout the study. In addition, a valve limited inspiratory flow to 0.5 L s^−1^ [19, 20]. The number of coughs elicited by each dose administration was counted for 15 s following each dose inhalation with the aid of an audio recorder (ICD-PX333, Sony Corporation, Tokyo, Japan) [19, 20]. The challenge test continued until ≥ 5 coughs were elicited by a single-dose administration. The threshold concentrations of capsaicin required to elicit 1 (C1), 2 (C2) and 5 (C5) coughs were calculated by interpolation [21].

## Cough Frequency Monitoring

Cough frequency was assessed objectively over 24 h with the Leicester Cough Monitor (LCM) [22]. The LCM is a validated ambulatory system, which comprises a MP3 recorder (ICD-PX333, Sony Corporation, Tokyo, Japan), a free-field lapel microphone (LFH9173, Philips, Amsterdam, Netherlands) and a semi-automated cough detection software. Coughs were detected as characteristic sounds, whether occurring in isolation or as part of a bout of prolonged coughing [22]. Total cough count (number of coughs over 24 h), mean cough frequency (coughs h^−1^ over 24 h), awake cough counts (number of coughs over estimated time spent awake) and awake 24-h cough frequency (coughs h^−1^) were recorded. Participants recorded and reported their time spent asleep.

## Subjective Assessments

### COPD-Specific Health Status

COPD health status was assessed with the COPD Assessment Test (CAT), a validated self-administered 8-item questionnaire (range 0–40; lower scores indicating better health status) [23]. The CAT has been demonstrated to be responsive to changes of health status during recovery following an exacerbation of COPD [24].

### Cough Severity and Cough-Specific Health Status

Cough severity was self-reported on a visual analogue scale (VAS) (range 0–100 mm; higher scores indicating more severe cough) [19]. The cough-specific health status was assessed with the Leicester Cough Questionnaire (LCQ), a self-administered 19-item assessment for cough-specific health status previously validated in COPD (range 3–21; higher scores indicating better health status) [25].

## Lung Function

Spirometry, body plethysmography and diffusing capacity of carbon monoxide (Jaeger MS-PFT Analyser Unit with Sentry Suite software version 2.19.96, Vyaire Medical, Mettawa, IL, USA) were measured as per guidelines by the ERS and the American Thoracic Society [26–28].

## Statistical Analysis

The distribution of data was assessed using the D’Agostino-Pearson test. Parametric data were summarised with means (standard deviation, SD), whilst non-parametric data with medians (interquartile range, IQR). Capsaicin challenge and cough frequency data were presented as geometric mean (geometric standard deviation, SD). Parametrically and non-parametrically distributed paired data were analysed with paired t test and the Wilcoxon matched-pairs signed rank test, respectively. Fisher’s exact test and Chi-squared test were used for categorical data. Correlations between variables were assessed with Pearson product-moment correlation coefficient (*r*) for parametric data, and Spearman’s rank-order correlation coefficient (*ρ*) for non-parametric data. Repeatability was analysed with the Bland–Altman method, and with intraclass correlation coefficients (ICC) based on a single-rater, absolute agreement, two-way mixed-effects model. *p* values < 0.05 were considered statistically significant with the exception for the analysis of factors associated with future exacerbation frequency when a Bonferroni correction was applied, whereby *p* values < 0.007 were considered statistically significant. The threshold concentrations of capsaicin required to elicit 1, 2 and 5 coughs (C1, C2 and C5, respectively) were calculated by interpolation of the log dose–response curve [21]. A value of 1000 µmol L^−1^ was assigned to any interpolated values which were > 1000 µmol L^−1^ [21].

A previous study has reported a significant reduction in capsaicin-evoked cough reflex sensitivity in 14 participants following recovery from an upper respiratory tract infection [13]. A study by Dicpinigaitis reported the reproducibility of the capsaicin cough challenge in a sample size of 10 subjects [20]. We therefore aimed to recruit 10 participants to investigate the repeatability of CRS in COPD, and 15 participants to investigate CRS during and following an exacerbation of COPD. All analyses were performed using Prism® version 8.1.1 (GraphPad Software, San Diego, California, USA), except the Bland–Altman and intraclass correlation correlations analyses which were performed with R version 3.6.1 (R Foundation for Statistical Computing, Vienna, Austria) for macOS version 10.14.5.

## Results

### Participant Characteristics

A total of 20 participants with exacerbation of COPD were recruited; demographics, anthropometrics and clinical characteristics are summarised in Table 1. The median (IQR) duration of hospital admission was 6 (3–8) days. Two participants required acute non-invasive ventilation; no participant required invasive mechanical ventilation or organ support. All participants were discharged from hospital to their usual place of residence, and had completed their course of oral corticosteroid before the second visit at 6 weeks. No participant took oral azithromycin during the course of this study. Seventeen out of 20 (90%) participants agreed to participate in the 12-month period of observation to determine subsequent exacerbation frequency (3 participants declined to participate). Ten separate participants with stable COPD were recruited to investigate the repeatability of cough reflex sensitivity testing and objective cough frequency (Table 2).

**Table 1:** COPD exacerbation group: demographics and clinical characteristics of participants during exacerbation and 6 weeks following recovery

	Participants with exacerbation of COPD (*n* = 20)		
	Admission	6-week recovery	*p* values
Age (years)	67.5 (6.3)	N/A	
Gender (female)	12 (60)	N/A	
BMI (kg m^−2^)	27.4 (7.3)	N/A	
Smoking status			> 0.999^c^
Ex	15 (75)	15 (75)	
Current	5 (25)	5 (25)	
MRC dyspnoea scale (stable state)	N/A	4 (3–5)	
CAT	30 (26–33)	27 (21–29)	0.039^d^
Spirometry			
FEV_1_% predicted	34.0 (10.1)	43.5 (15.0)	0.004^d^
FVC % predicted	65.4 (15.5)	83.8 (15.0)	< 0.001^d^
Body plethysmography^a^	N/A		
TLC % predicted		122.9 (108.0–130.4)	
RV % predicted		155.5 (39.1)	
Diffusion capacity^b^	N/A		
TL_CO_ % predicted		52.3 (16.7)	
K_CO_ % predicted		59.9 (23.9)	
GOLD stage*	N/A		
GOLD 1		0 (0)	
GOLD 2		5 (25)	
GOLD 3		11 (55)	
GOLD 4		3 (15)	
Co-morbidities		N/A	
Ischaemic heart disease	5 (25)		
Cerebrovascular disease	2 (10)		
Hypertension	11 (55)		
Diabetes mellitus	4 (20)		
Index exacerbation		N/A	
Duration of exacerbation symptom pre-admission (days)	3 (2–9)		
Systemic steroids use pre-admission	9 (45)		
Antibiotics use pre-admission	9 (45)		
Admission vital signs		N/A	
Temperature (°C)	37.0 (0.5)		
Heart rate (min^−1^)	96 (13)		
Respiratory rate (min^−1^)	21 (3)		
SpO_2_ (%)	94 (89–95)		
Routine laboratory test on admission		N/A	
Leucocytes (× 10^9^ L^−1^)	10.89 (3.96)		
Neutrophils (× 10^9^ L^−1^)	8.06 (3.65)		
Eosinophils (× 10^9^ L^−1^)	0.24 (0.21)		
Haemoglobin (g L^−1^)	140 (15)		
C-reactive protein (mg L^−1^)*	40 (69)		
Duration of admission (days)	6 (3–8)	N/A	

Data presented as mean (SD), median (IQR) or absolute values (percentage)

*BMI* body mass index, *MRC* Medical Research Council, *CAT* COPD Assessment Tool, *FEV*_*1*_ forced expiratory volume in 1 s, *FVC* = forced vital capacity, *TLC* total lung capacity, *RV* residual volume, *TL*_*CO*_ carbon monoxide transfer factor, *K*_*CO*_ carbon monoxide transfer coefficient, *GOLD* Global Initiative for Chronic Obstructive Pulmonary Disease, *SpO*_*2*_ transcutaneous oxygen saturation

*n = 19

^a^*n* = 13

^b^*n* = 16

^c^Fisher’s exact test

^d^Paired *t* test

**Table 2:** Stable COPD group: demographics and clinical characteristics of participants undergoing repeatability of cough measurements

	Participants with chronic obstructive pulmonary disease (*n* = 10)
Age (years)	64.0 (60.5–71.8)
Gender (female)	3 (30%)
BMI (kg m^−2^)	28.3 (19.8–34.4)
Smoking status	
Ex	7 (70)
Current	4 (40)
MRC dyspnoea scale	4 (3–4)
GOLD stage	
GOLD 1	2 (20)
GOLD 2	1 (10)
GOLD 3	6 (60)
GOLD 4	1 (10)
Lung function	
FEV_1_% predicted	45.7 (38.9–77.3)
FVC % predicted	97.1 (66.9–108.2)
FEV_1_/FVC (%)	38.5 (33.8–57.4)
TLC % predicted*	130.0 (102.2–138.6)
RV % predicted*	130.4 (92.3–183.9)
TL_CO_ % predicted^a^	43.5 (30.5–57.9)
K_CO_ % predicted^a^	49.5 (39.8–71.5)
CAT	25 (17–29)
LCQ	
Physical	5.5 (4.3–6.4)
Psychological	5.3 (4.5–7.0)
Social	5.4 (4.7–7.0)
Total	16.2 (13.4–20.5)

Data presented as median (IQR) or absolute value (percentage) unless otherwise stated

*BMI* body mass index, *MRC* Medical Research Council, *GOLD* Global Initiative for Chronic Obstructive Pulmonary Disease, *FEV*_*1*_ forced expiratory volume in 1 s, *FVC* forced vital capacity, *TLC* total lung capacity, *RV* residual volume, *TL*_*CO*_ carbon monoxide transfer factor, *K*_*CO*_ carbon monoxide transfer coefficient, *CAT* COPD Assessment Test, *LCQ* Leicester Cough Questionnaire

*n = 7

^a^*n* = 9

## Exacerbation of COPD

### Cough Reflex Sensitivity

**Fig. 1:**
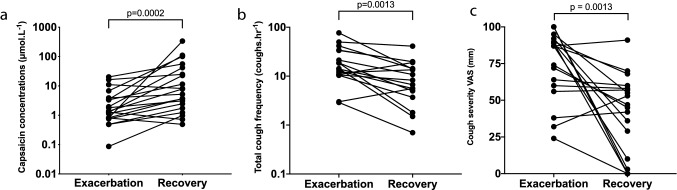
Capsaicin concentrations required to elicit 5 coughs (C5) during capsaicin challenge test, objective 24-h cough frequency and cough severity during and 6 weeks after exacerbation of chronic obstructive pulmonary disease. **a** C5 during and 6 weeks after exacerbation of COPD. **b** Total 24-h cough frequency during and 6 weeks after exacerbation of COPD. **c** Cough severity VAS during and 6 weeks after exacerbation of COPD

Cough reflex sensitivity was heightened during exacerbation compared to that following recovery. The capsaicin cough thresholds were significantly lower during exacerbation (*T*_E_) than at 6 weeks after recovery (*T*_R_) (Table 3). The geometric mean (SD) C5 at *T*_E_ and *T*_R_ were 1.76 (3.73) vs. 8.09 (6.25) μmol L^−1^, respectively, mean difference (95% CI) 2.20 (1.01–3.14) doubling doses (*p* < 0.001) (Fig. 1). The mean difference in C2 between *T*_E_ and *T*_R_ was 1.46 (*p* = 0.001) doubling doses (Table 3). There were no adverse events observed or reported by patients associated with capsaicin cough challenge testing during acute exacerbation of COPD. There was no significant difference in the change in C1, C2 and C5 doubling dose from *T*_E_ to *T*_R_ between gender (*p* = 0.392, 0.640 and 0.624, respectively). In addition, there was no association between the change in C5 doubling dose from *T*_E_ to *T*_R_, and age (*p* = 0.684), FEV_1_ (*p* = 0.333) and history of exacerbation of COPD prior to the index exacerbation (*p* = 0.211). There were no adverse incidents associated with capsaicin challenge tests performed during acute exacerbations in participants with COPD.

**Table 3:** Cough reflex sensitivity, objective cough frequency and subjective assessments during and 6 weeks after exacerbation of chronic obstructive pulmonary disease

	Admission	6-week recovery	*p* values
Cough reflex sensitivity*			
C1 (μmol L^−1^)	0.90 (3.48)	2.08 (3.51)	0.002^b^
C2 (μmol L^−1^)	1.06 (3.47)	2.92 (3.64)	0.001^b^
C5 (μmol L^−1^)	1.76 (3.73)	8.09 (6.25)	< 0.001^b^
24-h cough monitoring*,^a^			
Total cough count (coughs)	391.2 (2.4)	176.3 (2.9)	0.001^b^
Total cough frequency (coughs h^−1^)	16.3 (2.4)	7.4 (2.9)	0.001^b^
Awake cough count (coughs)	322.1 (2.3)	135.0 (3.2)	0.002^b^
Awake cough frequency (coughs h^−1^)	19.2 (2.2)	8.7 (3.2)	0.003^b^
Cough severity VAS (mm)^c^	74 (24)	40 (28)	0.001^d^
LCQ			
Physical	3.8 (1.2)	4.6 (1.3)	0.088^d^
Psychological	4.1 (1.6)	4.7 (1.4)	0.145^d^
Social	4.2 (1.8)	4.8 (1.7)	0.342^d^
Total	12.1 (4.3)	14.1 (4.1)	0.162^d^

Data presented as mean (SD) or median (IQR) unless otherwise stated

*C1* capsaicin concentration required to elicit 1 cough, *C2* capsaicin concentration required to elicit 2 coughs, *C5* capsaicin concentration required to elicit 5 coughs, *VAS* visual analogue scale, *LCQ* Leicester Cough Questionnaire

*Geometric mean (SD)

^a^*n* = 17

^b^Wilcoxon matched-pairs signed rank test

^c^*n* = 18

^d^Paired *t* test

### Objective Cough Frequency

Seventeen participants with exacerbation of COPD agreed to undergo 24-h cough monitoring. The geometric mean (SD) total 24-h cough frequency and awake cough frequency at *T*_E_ were 16.3 (2.4) and 19.3 (2.3) coughs h^−1^, respectively (Table 3). There was a significant reduction in total 24-h cough frequency between *T*_E_ and *T*_R_, mean difference (95% CI) 2.22 (1.43–3.44) fold difference (*p* = 0.001) (Fig. 1 and Table 3). There was no significant association between the changes in total 24-h cough frequency and changes in cough reflex sensitivity values from *T*_E_ to *T*_R_ (C1: *ρ* = 0.321, *p* = 0.209; C2: *ρ* = 0.355, *p* = 0.162 and C5: *ρ* = 0.436, *p* = 0.082). Neither was there a significant association between total 24-h cough counts and cough reflex sensitivity (C1, C2 and C5) at *T*_E_ (*ρ* = 0.147, 0.147 and 0.011, respectively, all *p* > 0.50).

### Subjective Measures

There was a significant reduction in median (IQR) CAT symptom scores at *T*_R_ compared to *T*_E_: 27 (21–29) vs. 30 (26–33), respectively (*p* = 0.039). There was no significant association between CAT scores and cough reflex sensitivity (C1, C2 and C5) at T_E_ (Table E1), or between the changes in CAT scores and cough reflex sensitivity (C1, C2 and C5) from *T*_E_ to *T*_R_ (*p* = 0.569–0.953).

There was a significant reduction in cough severity at *T*_R_ compared to *T*_E_ (mean (SD) cough severity VAS scores 74 (24) vs. 40 (28) mm, respectively (*p* = 0.001); Fig. 1) and a trend towards improvement in LCQ cough health status between these timepoints (mean (SD) LCQ total scores 14.1 (4.1) vs. 12.1 (4.3), respectively, *p* = 0.162). There was no significant association between cough reflex sensitivity (C1, C2 and C5) and either cough severity or LCQ cough-specific health status scores at *T*_E_ (Table E1), or between change in cough reflex sensitivity (C1, C2 and C5) and change in either cough severity or LCQ cough health status from *T*_E_ to *T*_R_ (*p* = 0.549–0.814, and *p* = 0.528–0.956, respectively).

## Association Between Cough Reflex Sensitivity and Future Exacerbation Frequency

Subsequent exacerbations of COPD occurred in 15 of the 17 individuals who were followed up for 12 months following the initial hospitalisation. The mean (SD) number of further COPD exacerbations per participant during follow-up was 2.5 (2.0) episodes. There were a total of 42 exacerbation episodes, of which 30 led to utilisation of primary care services, 12 to presentation to the emergency department, and 9 to hospital admission. The median (IQR) time to the next exacerbation following recruitment at *T*_E_ was 135 (78–238) days.

**Fig. 2:**
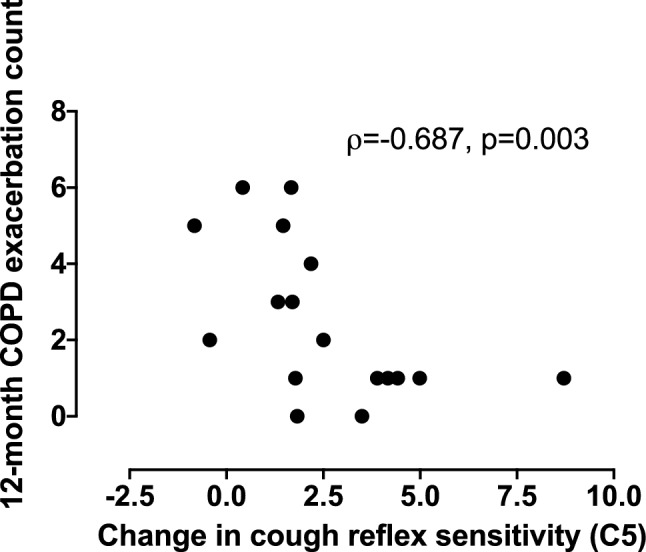
The relationship between the change in cough reflex sensitivity following exacerbation of chronic obstructive pulmonary disease and future exacerbations

**Fig. 3:**
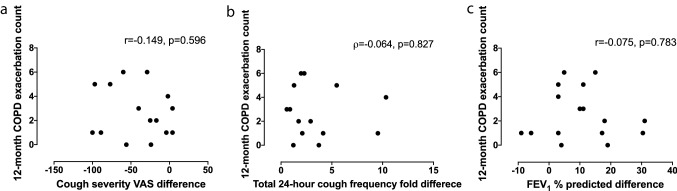
The relationship between COPD exacerbation frequency and change between exacerbation and 6-week recovery visit in cough severity visual analogue scale score, objective cough frequency and forced expiratory volume in 1 s. **a** Cough severity visual analogue scale. **b** Total (24-h) cough frequency. **c** Forced expiratory volume in 1 s

There was a significant association between the change in C5 from *T*_E_ to *T*_R_ and the rate of acute exacerbation of COPD in the following 12 months (*ρ* = − 0.687, *p* = 0.003) (Table 4 and Fig. 2). In addition, there was no significant association between the rate of exacerbation of COPD in the following 12 months and the changes from *T*_E_ to *T*_R_ in either objective cough frequency, CAT scores (*p* = 0.116), cough severity VAS (*p* = 0.596), LCQ or FEV_1_% predicted (Table 4 and Fig. 3). Neither at time *T*_E_ or *T*_R_ was there an association between the rate of exacerbation of COPD in the following 12 months and CRS, objective cough frequency, CAT scores, cough severity VAS, LCQ or FEV_1_% predicted (all *p* > 0.06).

**Table 4:** Relationship between cough and 12-month prospective frequency of exacerbation of chronic obstructive pulmonary disease

	Future 12-month exacerbation count	
	Correlation coefficients	*p* values
Admission vs. 6-week recovery		
Cough reflex sensitivity (C5)*	− **0.687**	**0.003**
Total cough count (coughs)^a,b^	− 0.064	0.827
Awake cough count (coughs)^a,b^	0.033	0.912
LCQ	0.244^c^	0.345
FEV_1_% predicted	− 0.075^c^	0.783

All correlation coefficients are Spearman’s rank-order correlations unless stated otherwise. Bonferroni correction was applied and *p* values < 0.007 were considered significant

Significant *p* values are given in bold

*C5* capsaicin concentration required to elicit 5 coughs, *VAS* visual analogue scale, *LCQ* Leicester Cough Questionnaire, *CAT* COPD Assessment Test, *FEV*_*1*_ forced expiratory volume in 1 s

*Doubling dose change

^a^*n* = 15

^b^Fold difference

^c^Pearson product-moment correlation

## Repeatability of Cough Reflex Sensitivity and Objective Cough Frequency in Stable COPD

**Fig. 4:**
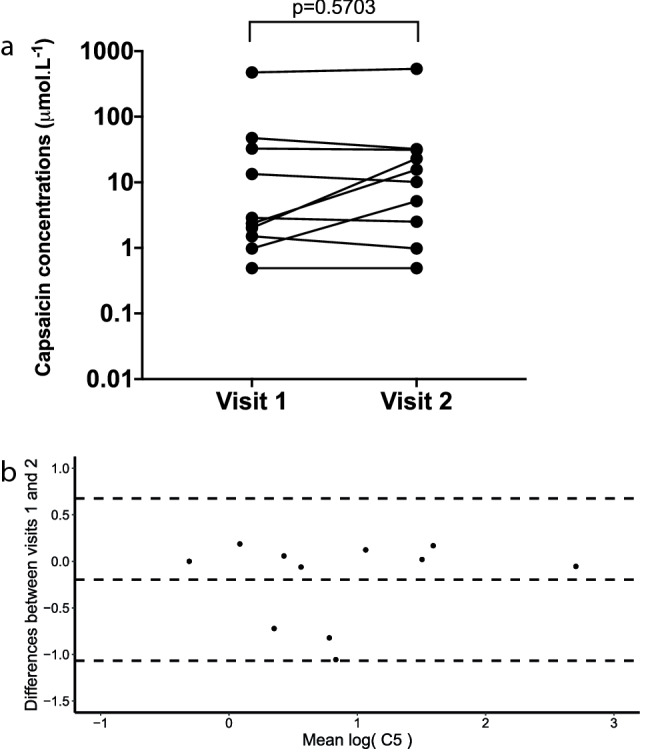
Repeatability of capsaicin cough challenge test threshold to elicit 5 coughs (C5) during capsaicin challenge test in stable chronic obstructive pulmonary disease. **a** C5 (6 weeks apart) in stable COPD. **b** Bland–Altman plot of C5 (6 weeks apart) in stable COPD

No participant was excluded from the study of CRS repeatability. Intraclass correlation coefficients to determine repeatability of the cough reflex sensitivity measures log(C1), log(C2) and log(C5) were 0.37, 0.54 and 0.85, respectively, in participants with stable COPD (Table 5). The mean differences (95% CI) in C1, C2 and C5 over 6 weeks were 0.34 (− 0.13–2.40), 0.31 (− 0.11–2.16) and 0.21 (− 0.40–1.79) doubling doses, respectively (Fig. 4). C5 was the most repeatable capsaicin challenge test threshold and was therefore used for the main analysis.

**Table 5:** Repeatability of capsaicin challenge test, objective cough frequency and cough severity over 6 weeks in participants with stable chronic obstructive pulmonary disease

	Week 1	Week 6	ICC
Cough reflex sensitivity			
C1 (μmol L^−1^)	0.93 (2.89)	2.04 (3.90)	0.37
C2 (μmol L^−1^)	1.50 (3.14)	3.05 (4.13)	0.54
C5 (μmol L^−1^)	6.29 (8.44)	10.20 (7.45)	0.85
24-h cough monitoring*			
Total cough count (coughs)	124.7 (4.1)	165.0 (2.6)	0.86
Total cough frequency (coughs h^−1^)	5.1 (4.2)	6.9 (2.5)	0.85
Awake cough count (coughs)	101.5 (3.9)	134.0 (2.4)	0.70
Awake cough frequency (coughs h^−1^)	6.5 (3.9)	8.4 (2.4)	0.84
Cough severity VAS (mm)	37 (23)	45 (30)	0.56

Data presented as geometric mean (SD) or mean (SD)

*ICC* intraclass correlation coefficient, *C1*capsaicin concentration required to elicit 1 cough, *C2* capsaicin concentration required to elicit 2 coughs, *C5* capsaicin concentrations required to elicit 5 coughs, *VAS* visual analogue scale

*n = 9

**Fig. 5:**
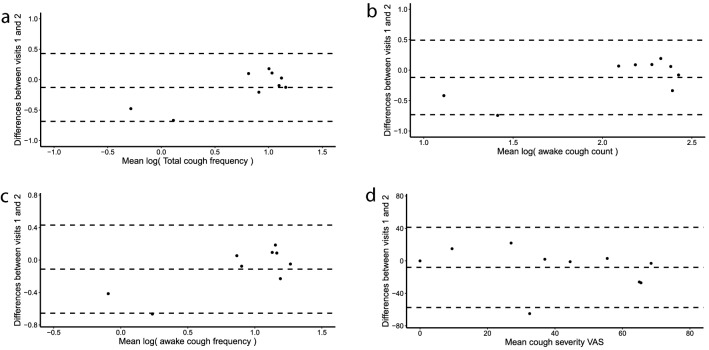
Bland–Altman plot assessing the repeatability of threshold capsaicin concentrations during capsaicin challenge test, objective cough frequency and cough severity visual analogue scale over 6 weeks in participants with stable chronic obstructive pulmonary disease. **a** Total cough frequency. **b** Awake cough count. **c** Awake cough frequency. **d** Cough severity visual analogue scale

For objective cough frequency, the ICC coefficients for repeatability of log(total 24-h cough count), log(total cough frequency), log(awake cough count) and log(awake cough frequency) over 6 weeks were 0.86, 0.85, 0.70 and 0.84, respectively (Table 5). The mean differences (95% CI) in total cough frequency, awake cough count and awake cough frequency were 0.76 (0.47–1.22), 1.32 (0.76–2.29) and 0.11 (0.79–2.12) fold differences, respectively (Fig. 5). The ICC for repeatability of cough severity VAS scores over 6 weeks was 0.56 (Table 5 and Fig. 5).

## Discussion

We investigated the sensitivity of the cough reflex during exacerbations of COPD and its relationship with the frequency of future exacerbations. Cough reflex sensitivity (CRS) was heightened during exacerbations of COPD. The magnitude of change in cough reflex sensitivity 6 weeks after hospitalisation for exacerbation was associated with the frequency of future exacerbations. There was no association between subjective cough severity, objective cough frequency or FEV_1_ and the frequency of exacerbations. In stable COPD patients, C5 was the most repeatable measure of CRS.

To the best of our knowledge, our study is the first to investigate CRS during exacerbations of COPD. CRS to capsaicin was heightened during exacerbation. Viral upper respiratory tract infections are commonly associated with exacerbations of COPD [29]. There are studies which have investigated CRS in healthy subjects with acute cough associated with upper respiratory tract infection [13, 14]. O’Connell et al. demonstrated that CRS was heightened during upper respiratory tract infection, and reduced following 4 weeks of recovery in healthy participants with non-productive cough [13]. Dicpinigaitis et al. also reported that CRS to capsaicin was heightened during upper respiratory tract infection, and reduced following 4 weeks of recovery [14]. Capsaicin is a selective agonist of transient receptor potential vanilloid 1 ion channel receptors (TRPV1), which are expressed on C-fibre sensory nerves [30]. Bronchopulmonary C fibres are relatively quiescent in health and can be sensitised or activated by inflammatory mediators [11]. Exposure to ozone [12], lactic acid [12], cationic proteins [31] and prostaglandin-E_2_ [11] are known to enhance pulmonary C-fibre responses to mechanical and chemical stimuli in a reversible fashion. Airway inflammation in exacerbation of COPD could therefore lead to sensitisation or activation of C fibres.

A key finding from our study was that the magnitude of change in CRS following an exacerbation was associated with prospective frequency of exacerbations. The frequency of exacerbation in our study was comparable to those in previous studies [2, 17]. Further exacerbations were more frequent in individuals demonstrating a lower reduction in cough reflex sensitivity at six weeks after the index episode. Previous studies have investigated the predictive factors for exacerbations of COPD, including exacerbation frequency in the preceding 12 months, disease severity, gastro-oesophageal reflux and health status [17, 32]. A failure of CRS to change during recovery from exacerbation has not been previously studied as a potential marker of future exacerbation risk. Terada et al. conducted the only study of CRS and its association with exacerbations, and investigated CRS in patients with stable COPD [33]. In contrast to our findings, Terada et al. did not find an association between CRS and exacerbation of COPD defined by a change of medication [33]. There are several key differences between the studies that may explain the contrasting findings. Terada et al. assessed a single measure of CRS during a stable phase and did not assess it longitudinally for the persistence of CRS following exacerbation of COPD [33]. Terada et al. did however report a weak correlation with frequency of exacerbations of COPD when defined on the basis of a change of symptoms rather than a change in therapy for exacerbation of COPD [33].

A strength of the current study is that measurement of the change in CRS within the same individual at two time points controls for much of the large variation in cough reflex sensitivity that we have confirmed to exist between subjects in the stable state in COPD*.* This large inter-individual variation in CRS might explain the apparent lack of association between absolute values of C5 and COPD exacerbation frequency.

The mechanism by which a persistently heightened cough reflex sensitivity may predispose to future exacerbations is not clear. An associated persistence of airway inflammation following an exacerbation may be important but this has seldom been studied. Airway inflammation in patients with stable COPD however has been reported to predispose to exacerbations. Bhowmik et al. demonstrated that raised levels of cytokines, IL-6 and 8, in sputum were associated with frequent exacerbations [34]. Gompertz et al. reported reduced levels of sputum protease inhibitors in those with frequent COPD exacerbations [35]. Another possible mechanism for persistent cough reflex sensitivity, and predisposition for future exacerbation and symptoms, is changes in central neural pathways and cognitive influences, such as those that have been reported in inflammatory airway diseases such as asthma using functional neuroimaging [36].

The assessment of CRS may be a tool to identify patients at high risk of future exacerbations. The existing cough challenge methodology is not practical for routine clinical use and simpler protocols would need to be developed. The capsaicin challenge test was however well tolerated by severe COPD patients suffering from acute exacerbation study. This is the first study to assess cough reflex sensitivity during an exacerbation and may facilitate future research investigating the mechanisms involved in exacerbation of COPD. It is not possible to elucidate the mechanism between the magnitude of change in CRS following exacerbation and the frequency of future exacerbations from our study. A better understanding of this mechanism may lead to targeted therapy for the prevention of exacerbations.

Previous studies have investigated whether other measures of cough predict COPD exacerbation frequency, with conflicting findings. Burgel et al. and Seemungal et al. reported that the presence of self-reported cough is associated with exacerbation frequency in stable COPD [2, 7]. In contrast, Hurst et al.[17], in common with this current study, did not find an association between self-reported cough and numbers of exacerbation of COPD. Neither did we observe an association between objective cough counts and exacerbation frequency. Objective CRS, subjective symptom scales and objective cough counts assess very different dimensions of cough, and a poor association between them is well reported [37]. It is likely that CRS reflects the interaction between airway inflammation and sensory nerves.

There are limitations to our study. We studied a relatively small sample size and were therefore unable to assess the predictors of exacerbations of COPD with multivariate regression analysis. Our patients had severe COPD and therefore our findings should be interpreted with caution for those with mild disease. We were also unable to assess the effects of factors such as smoking status that may influence CRS due to the small sample size. To standardise the timing of the cough challenge test during the hospital admission, patients were assessed on the first day that they were deemed fit for a challenge test by the clinical and research teams. It is possible that the variability of the timing of the cough challenge test between the patients may have impacted the cough reflex sensitivity assessment. The assessment of CRS in the recovery phase may have been too soon after the exacerbation. We did, however, demonstrate that CRS is highly repeatable over 6 weeks in stable COPD with no order effect. Furthermore, the CRS values following recovery from exacerbation in our study were similar to those reported previously in stable COPD by Sumner et al.[38]*.* FEV_1_% predicted during exacerbation, following recovery and the change between 2 occasions were not associated with the frequency of future exacerbations despite previous reports of an association between severity of COPD according to FEV_1_% predicted and exacerbation frequency [17, 39]. This may be due to the small sample size and the relatively limited range of disease severity in our study. Patients with more severe disease were more likely to have exacerbations, and therefore be included in our study. The subjective cough measures were not associated with CRS, which may be due to the small sample size in our study. This is not unexpected as health status and CRS assess very different dimensions of cough, and is consistent with previous reports [37, 40]. We did not find an association between cough reflex sensitivity and objective cough frequency, and this is consistent with a previous study reporting a poor association [38].

The technology for measuring cough frequency in the current study has not yet been widely used in COPD, but is well established in chronic cough in other contexts [40]. Validity for our approach though is suggested by the similar magnitude and range of cough frequencies we report in stable COPD compared to those of a study by Sumner et al. using an alternative method to determine cough frequency [38]. Whilst there was a slight excess of female participants in our cohort, there was no significant difference in C5 between gender. A slight excess of female participants is consistent with other studies of patients hospitalised for exacerbation of COPD [16, 41]. Whilst C5 generally appeared a repeatable measure of cough reflex sensitivity, this observation was not universal; some participants with stable COPD demonstrated marked differences in C5 between the two timepoints. This may therefore impact the utility of the C5 test in future studies. We did not investigate the cause of COPD exacerbation or markers of inflammation in sputum and serum, and these should be assessed in future studies. We only assessed CRS with inhaled capsaicin. It is possible that COPD patients may respond differently to other tussive agents, and produce different results [42, 43].

In conclusion, cough reflex sensitivity is heightened during acute exacerbation of COPD and is associated with the frequency of future exacerbations. Further studies are needed to replicate our findings in a larger study, and to investigate the mechanisms for persistent cough reflex hypersensitivity as this may lead to targeted therapy for the prevention of COPD exacerbations.

## Electronic Supplementary Material

Below is the link to the electronic supplementary material.

Supplementary file1 (DOCX 18 kb)
