# Supplementary material for: The Relationship Between Cough Reflex Sensitivity and Exacerbation Frequency in Chronic Obstructive Pulmonary Disease
Source: Lung. 2020 Jun 19;198(4):617–28. doi: 10.1007/s00408-020-00366-x (PMC7374441; doi:10.1007/s00408-020-00366-x)
Supplement: Supplementary file 1 — Supplementary file1 (DOCX 18 kb) [file 408_2020_366_MOESM1_ESM.docx]

# Table E1. The Relationship of capsaicin threshold concentrations from capsaicin challenge test, chronic obstructive pulmonary disease health status, cough severity and cough-specific health status

|  | **C1 μmol⋅L^-1^** | | **C2 μmol⋅L^-1^** | | **C5 μmol⋅L^-1^** | |
| --- | --- | --- | --- | --- | --- | --- |
|  | **Correlation coefficients** | **p value** | **Correlation coefficients** | **p value** | **Correlation coefficients** | **p value** |
| **CAT** | -0.158 | 0.518 | -0.158 | 0.518 | -0.104 | 0.671 |
| **Cough severity VAS (mm)** | 0.168 | 0.504 | 0.168 | 0.504 | -0.023 | 0.927 |
| **LCQ** |  |  |  |  |  |  |
| Physical | 0.138 | 0.562 | 0.138 | 0.562 | 0.200 | 0.399 |
| Psychological | 0.159 | 0.502 | 0.159 | 0.502 | 0.240 | 0.308 |
| Social | 0.078 | 0.745 | 0.078 | 0.745 | 0.189 | 0.424 |
| Total | 0.068 | 0.774 | 0.068 | 0.774 | 0.180 | 0.448 |

All correlation coefficients are Spearman’s rank-order correlation.

C2 and C5 = capsaicin threshold concentrations required to elicit 2 and 5 coughs respectively; VAS = visual analogue scale; LCQ = Leicester Cough Questionnaire; CAT = COPD Assessment Test.
